# Combination of ozone and ultrasonic-assisted aerosolization sanitizer as a sanitizing process to disinfect fresh-cut lettuce

**DOI:** 10.1016/j.ultsonch.2021.105622

**Published:** 2021-06-06

**Authors:** Jiayi Wang, Yangyang Zhang, Yougui Yu, Zhaoxia Wu, Hongbin Wang

**Affiliations:** a College of Food and Chemical Engineering, Shaoyang University, Shaoyang 422000, China; b Shijiashike Co., Ltd., Liaoyang 111000, China; c College of Food Science, Shenyang Agricultural University, Shenyang 110866, China

**Keywords:** Produce disinfection, Gaseous ozone, Aerosolization sanitizer

## Abstract

Reduction of sanitizer dosage and development of non-immersion disinfection methods have become major focuses of research. Here, we examined the disinfection efficacy of combining gaseous ozone (4 and 8 ppm) with aerosolized oxidizing sanitizer [sodium hypochlorite (SH, 100 and 200 ppm)] and aerosolized organic acid [acetic acid (AA, 1% and 2%) and lactic acid (LA, 1% and 2%)]. Notably, 1% AA and 4 ppm gaseous ozone were ineffective for disinfecting *Salmonella* Typhimurium, and treatment with 1% AA + 8 ppm ozone caused browning of lettuce leaves and stimulated increases in aerobic mesophilic count (AMC), aerobic psychrotrophic count (APC), *S.* Typhimurium, and *Escherichia coli* O157:H7. Treatment with 2% LA + 8 ppm ozone resulted in the lowest *S.* Typhimurium, *E. coli* O157:H7, *Listeria monocytogenes*, AMC, APC, and molds and yeasts during storage (0–7 days at 4 °C). Quality analysis indicates that LA + 8 ppm ozone and SH + 8 ppm ozone did not negatively affect L*, a*, b*, polyphenolic content, weight loss, and sensory properties; however, the levels of two individual phenolic compounds (3,4-dihydroxybenzoic acid and vanillin), responsible for phenylpropanoid synthesis, were significantly increased after treatment with 2% LA + 8 ppm ozone. These findings provided insights into the use of LA combined with gaseous ozone for application in disinfecting fresh produce.

## Highlights

• Combination of ozone and ultrasonic-assisted aerosolization sanitizer was investigated to disinfect fresh-cut lettuce.
• Quality properties were not negatively affected by ozone + lactic acid and ozone + sodium hypochlorite.
• Combination of 8 ppm ozone and 2% lactic acid was most effective to control microbial growth.

## 1 Introduction

The US Food and Drug Administration (FDA) recommends consuming 3–5 different vegetables and 2–4 different fruits every day [1]. Minimally processed produce has the characteristics of convenience and variety, which cater to people's increasingly fast-paced lifestyle. However, because fresh-cut vegetables are generally eaten in raw form without heat treatment, the risk of microbial contamination increases. Microbial contamination causes spoilage, shortens shelf life, and results in food-borne diseases [2]. Owing to the short shelf life of fresh-cut produce, food-borne diseases caused by food-borne pathogens are the biggest food safety hazards [3]. The pathogens that often cause foodborne disease related to the consumption of fresh produce are *Escherichia coli* and *Salmonella* spp., accounting for 30.87% and 47.65% of cases in the United States of America and 8.33% and 47.62% of cases in Europe, respectively [4]. The contamination of fresh produce by foodborne pathogens is also serious in developing countries. For example, in Brazil, 53.1% and 3.7% of ready-to-eat vegetables were found to be contaminated with *E. coli* and *Listeria monocytogenes*, respectively [5]. Additionally, in Rwanda, 6.1%, 5.1%, and 1% of farm vegetables were reported to be contaminated with *E. coli*, *Salmonella* spp., and *L. monocytogenes*, respectively [6]. Therefore, it is necessary to adopt low-cost disinfection methods that do not negatively affect the quality of fresh produce.

In recent years, technologies such as microbe-microbe interactions, pulsed light, and cold plasma have been widely studied as emerging nonthermal disinfection technologies [7], [8], [9]. Although these approaches have many advantages in the context of industrialization, they are generally not applied on a large scale, owing to the high cost of the equipment. However, chemical sanitizers have the advantages of low cost, good disinfection, and ability to mix with water at different ratios, enabling their broad application [10]. Pathogenic bacterial infections often occur on some vegetable leaves. When fresh-cut vegetables are washed, the wash water is circulated; thus, when infected vegetable leaves are placed into the washing tank, the pathogen will enter the circulated wash water. Subsequently, when uninfected leaves are placed into the washing tank, cross-contamination can occur, leading to infection of all vegetables and increasing the risk of foodborne diseases [11], [12], [13]. Additionally, reduction of sanitizer dosage is required to better meet the cost requirements of minimal processing industries [14], [15]. Therefore, reduction of sanitizer dosage and development of non-immersion disinfection methods have become major focuses of research.

Many recent studies have explored the minimum free chlorine (FC) concentration to prevent cross-contamination during fresh produce washing. For example, Luo et al.[16] found that maintaining at least 10 mg/L FC at industry scale can strongly reduce the likelihood of bacterial survival in the wash water. Additionally, Gómez-López et al.[13] found that 7 mg/L FC is an effective concentration for inactivating *E. coli* O157:H7 in wash water. However, few reports have described disinfection methods that can simultaneously minimize sanitizer dosage and meet non-immersion characteristics (i.e., no immersion in aqueous sanitizers). In addition, changes in microbial growth and fresh produce quality after washing can only be controlled by packaging, storage environment, coatings, and microbe-microbe interactions. However, during practical application, microbe-microbe interactions and coating film methods are complicated and expensive.

Ultrasonic-assisted aerosolization has the characteristics of consuming a low amount of sanitizer and the formation of micron-sized particles, which can attach to the leaf surface and continuously control the microbe levels after treatment. Among all aqueous sanitizers, the disinfection efficacy and cost of chemical disinfectants are most suitable for practical application [9], [17]. Chemical sanitizers can be divided into two types, based on their antibacterial mechanism of action: organic acids and oxidizing sanitizers [10]. Among chemical sanitizers, the oxidizer ozone and chlorine-based sanitizers are commonly used, owing to their moderate efficiency and extremely low cost [12], [18]. For example, ozone can degrade pesticide residues, disinfect microbes on the surface of the produce, and be prepared using air, and therefore, only require low equipment cost [10], [19]. However, use of chlorine sanitizers to disinfect fresh produce is prohibited in some countries, such as Germany, Switzerland, Netherlands, Belgium, and Singapore [7], [20], [21]. As another type of chemical sanitizer, most organic acids are food additives with a high food safety level and are approved as Generally Recognized as Safe (GRAS) by the FDA. Among them, acetic acid (AA) and lactic acid (LA) exhibit higher disinfection effects and relatively lower cost compared with other GRAS organic acids (e.g., tartaric acid, succinic acid, and propionic acid) [1], [22]. According to previous reports, generally, sodium hypochlorite (SH) is used at a concentration of 50–200 ppm, while AA and LA are used at a concentration of 0.5–2%, with the treatment time not allowed to exceed 5 min; this is mainly because high concentration and long processing time will cause quality deterioration of the produce [7], [18], [23]. The ozone concentration used for fresh produce processing is generally 0.5–10 ppm [24].

In this study, we aimed to evaluate the disinfection effects of combinations of gaseous ozone (GO, 4–8 ppm) and aerosolized chemical sanitizers (100–200 ppm SH, 1–2% AA, and 1–2% LA) using fresh-cut green leaf lettuce as a model.

## 2 Materials and methods

### 2.1 Sample preparation

Green leaf lettuce (*Lactuca sativa* L. var. crispa) was purchased from a local market on the day of the experiment. After rinsing for 30 s to remove dirt, the two outer leaves, inner baby leaves, and stems were removed, and a circle knife (diameter 5.2 × 10^−2^ m) was used to cut the sample [9], [10]. The obtained samples were drained using a manual salad spinner sterilized with 75% ethanol.

### 2.2 Pathogen inoculation

The inoculation procedure was carried out according to our previous report [9], [25]. Single colonies of *E. coli* O157:H7 (NCTC12900), *L. monocytogenes* (ATCC19115), and *Salmonella* Typhimurium (ATCC14028) were inoculated into nutrient broth (Hopebio, Qingdao, China) and shaken overnight at 37 °C. The bacterial suspension was adjusted to 10^9^ CFU/mL, and 5 mL of this culture was then added to a stomacher bag containing 200 mL sterilized 0.85% NaCl solution. Then, 10 g of the lettuce sample was placed into the bag and massaged for 20 min. The sample was then placed on a sterilized plastic tray in a biosafety cabinet and air dried.

### 2.3 Disinfection

The sanitizers used in this study were SH (Sinopharm, Beijing, China), AA (Macklin, Shanghai, China), and LA (Macklin). The concentration of FC was adjusted to 100 or 200 ppm using a DPD test kit (Lohand, Hangzhou, China). The concentrations of AA and LA were adjusted to 1% and 2%. The concentrations of GO were 4 and 8 ppm, respectively.

**Fig. 1:**
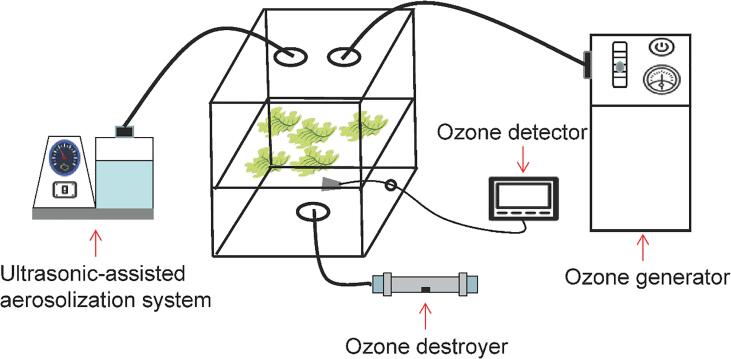
Schematic diagram of the disinfection equipment.

A schematic of the equipment used in this study is shown in Fig. 1. An acrylic chamber (50 cm × 50 cm × 60 cm) was used as a disinfection box, and the ultrasonic-assisted nebulizer (aerosolization rate and ultrasonic frequency: 3.6 mL/min and 1.7 MHz, respectively; 402AI, Yuwell, Shanghai, China) and ozone generator (10 g/h; Shenghuan, Guangzhou, China) were connected at the top of the box. The ozone concentration was detected using an ozone detection probe located under the sample carrying plate, and the probe was corrected using the KI method. When the box was filled with the aerosolized sanitizer and the ozone concentration was reached, we quickly pushed the lettuce sample into the box from the right side and then disinfected the sample for 3 min. After disinfection, the samples were transferred to plastic boxes, covered with plastic wrap, and stored at 4 °C for 7 days. Excess ozone was discharged from the bottom of the box and was thermally destroyed to oxygen using an ozone destroyer (Zoneche, Guangzhou, China).

### 2.4 Microbiological analysis

Samples were analyzed at 0, 3, and 7 days. A 25-g sample was homogenized with 225 mL sterile NaCl solution for 1.5 min in a stomacher bag. Then, the suspension was serially diluted. The suspension (0.1 mL) was surface-plated on modified sorbitol MacConkey agar (Hopebio), Listeria chromogenic agar (Land Bridge, Beijing, China), and xylose lysine deoxycholate agar (Hopebio) to analyze *E. coli* O157:H7, *L. monocytogenes*, and *Salmonella* Typhimurium, respectively, and incubated for 24 h at 37 °C. For naturally present microbes, 0.1 mL of the diluted bacterial suspension was surface-plated on Rose Bengal agar (Hopebio) and incubated at 30 °C for 3 days to quantify molds and yeasts (M&Y). In addition, 1 mL of the suspension was pour-plated onto plate count agar (Hopebio) and incubated at 7 °C for 10 days to obtain the aerobic psychrotrophic count (APC) and or at 37 °C for 2 days to obtain the aerobic mesophilic count (AMC). All results are expressed as log CFU/g.

### 2.5 Quality analysis

#### 2.5.1 Color analysis

At the end of the storage period (day 7), 10 leaves were randomly selected from each package for instrument color analysis. L*, a*, and b* values were detected at two locations per piece using a colorimeter (CR400; Konica Minolta, Osaka, Japan). Before use, the colorimeter was calibrated using a white standard plate (Y = 82.80, x  = 0.3194, y = 0.3264).

#### 2.5.2 Sensory analysis

Fifteen panelists (ages 25–40 years) from Liaoyang, Liaoning, China were invited to evaluate sensory color, flavor, and crispness. A 3-point scale method was used for evaluation, where 0 was very bad (not characteristic of the product), 5 was the acceptability threshold, and 10 represented very good product characteristics [10], [25]. The plates containing lettuce samples were marked on the bottom and reordered before analysis. The sensory room was equipped with a 40-W white light, and only one person was allowed to enter the room during the evaluation. For flavor evaluation, after evaluating one sample, the tester rinsed the mouth with drinking water three times and then evaluate the next sample after 30 s.

#### 2.5.3 Weight loss analysis

Weight loss during the storage period (0–7 days) was analyzed according to the following formula:

$$\mathrm{Weight}\;loss\left(\%=1-\frac{\mathrm{Weigh}t_{7d}}{\mathrm{Weigh}t_{0d}}\right)$$

#### 2.5.4 Polyphenolic content analysis

The content of polyphenols was analyzed at the end of storage (7 days) using the Folin-Ciocalteu method [26], with minor modifications. Ten grams of the sample was extracted using 150 mL of 80% methanol in a blender for 2 min. Then, the sample was incubated for 2 h at 4 °C to ensure sufficient extraction, and the homogenate was centrifuged at 12,000 rpm for 10 min to obtain supernatants. The supernatant (50 μL) was added to 3 mL distilled water, followed by the addition of 250 μL Folin-Ciocalteu reagent, and the reaction was allowed to occur for 6 min. Finally, 750 μL of 20% sodium carbonate was added to neutralize the reaction, and samples were incubated for 90 min in the dark. The absorbance was measured at 765 nm, and the results were expressed as gallic acid equivalents (GAE; mg/kg) on a fresh weight basis.

#### 2.5.5 Individual phenolic analysis

Targeted metabolomics was applied to analyze the individual phenolics at the end of storage (7 d). The group of 2% LA + 8 ppm ozone and control were selected. Standard solutions of individual phenolic compounds were prepared at ten different concentrations (0.1, 0.5, 1, 10, 50, 100, 150, 200, 300, and 500 ng/mL) using 50% methanol. The fresh sample was homogenized and 0.1 g of the resulting homogenate was extracted using 2 mL of 1 N NaOH for 2 h, followed by the addition of 0.5 mL of 5 M HCl. Then, 2 mL ethyl acetate was used to purify the sample in three rounds of purification, followed by blow drying using nitrogen. Subsequently, 0.5 mL of methanol was added to obtain a sample for liquid chromatography (LC)-electrospray ionization (ESI)-mass spectrometry (MS) analysis. UHPLC (Vanquish, Thermo, USA) system equipped with a Waters HSS T3 column (100 × 2.1 mm, 1.8 µm) was applied for analysis. Injection volume was 2 μL, and the column temperature was 40 °C. Mobile phase A was 0.1% formic acid (FA) with acetonitrile, and mobile phase B was 0.1% FA with water. The flow rate was 0.3 mL/min, and the gradient elution was as follows: 0–2 min, 10% A; 2–6 min, A was linearly increased from 10% to 60%; 6–8 min, 60% A; 8–8.1 min, A was linearly decreased from 60% to 10%; 8.1–10 min, 10% A. The separated sample was then subjected to a Q-exactive mass spectrometer (Thermo). ESI negative and single ion monitoring modes were used for detection. ESI source conditions were as follows: spray voltage, 3 kV; source temperature, 350 °C; full ms resolution, 70000; sheath gas flow rate, 40 Arb; aux gas flow rate, 10 Arb.

### 2.6 Statistical analysis

Differences between group means were evaluated using SPSS v.20 (SPSS, Chicago, IL, USA), and differences in mean values were analyzed using Duncan's multiple range tests. Results with P values of <0.05 were considered significant. All experiments were independently replicated three times.

## 3 Results and discussion

### 3.1 Screening of single treatment and aerosolization rate against *S.* Typhimurium on fresh-cut lettuce

**Fig. 2:**
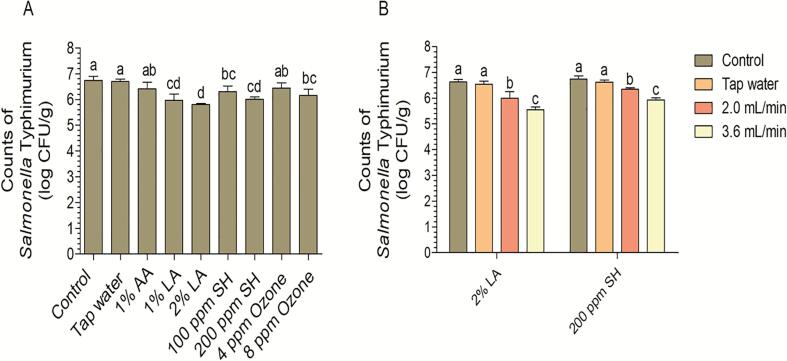
Counts of *Salmonella* Typhimurium (log CFU/g) on fresh-cut lettuce after disinfection with several aerosolized sanitizers (A) and different aerosolization rate (B). Bars show mean ± standard deviation values, and different letters above the columns indicate significant differences (P < 0.05). AA, acetic acid; LA, lactic acid; SH, sodium hypochlorite.

After storage for 7 days, browning spots were observed on samples treated with 2% AA; thus, 2% AA was not selected for the screening experiment. This phenomenon was also observed by Wang et al. [22], who found that washing with 1% AA for 1.5 min and storage for 5 days resulted in visual quality loss. As *Salmonella* spp. is the pathogen that causes the most foodborne diseases [4], sanitizer screening experiments were carried out against *S.* Typhimurium. The results indicated that 1% AA and 4 ppm ozone were ineffective at disinfecting *S.* Typhimurium (Fig. 2A). Practical application depends on the equipment available in the market, and most of the existing ultrasonic-assisted nebulizers have the frequency of 1.7 MHz (e.g. Yuwell®, Omron®, and Folee®); thus, an ultrasonic frequency of 1.7 MHz was used in this study. Aerosolization rate screening experiments indicated a disinfection effect at 3.6 mL/min (the maximum aerosolization rate was significantly higher than 2.0 mL/min; Fig. 2B); thus, 3.6 mL/min was used as the aerosolization rate in subsequent experiments. Because the objective of this study was to combine an aerosolized sanitizer with GO, we selected 8 ppm ozone in subsequent experiments. Moreover, no previous studies have reported whether the combination of 1% AA and 8 ppm ozone showed disinfection effects. Therefore, the combination of 1% AA and 8 ppm ozone was applied in subsequent experiments.

### 3.2 Disinfection efficacies of different combinations on fresh-cut lettuce

**Fig. 3:**
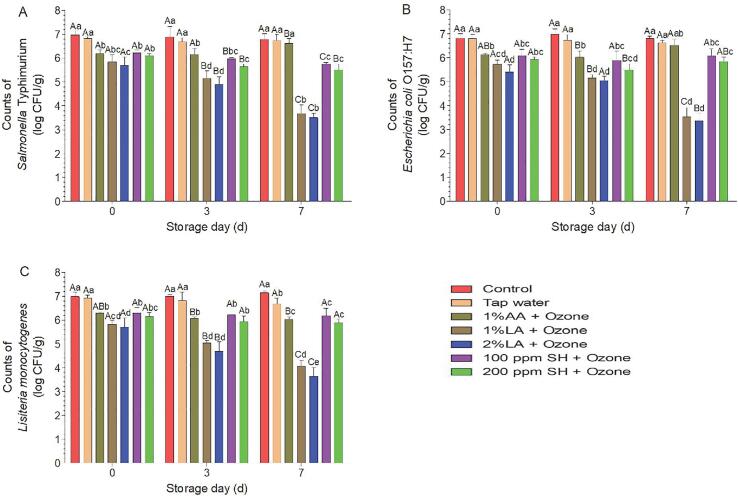
Disinfection effects against *S.* Typhimurium (A), *E. coli* O157:H7 (B), and *L. monocytogenes* (C) during storage. Within the same day, mean values with different lowercase letters are significantly different from each other (P < 0.05); within the same treatment, mean values with different capital letters are significantly different from each other (P < 0.05). AA, acetic acid; LA, lactic acid; SH, sodium hypochlorite. The concentration of ozone was 8 ppm.

Despite browning of lettuce leaves caused by 1% AA + 8 ppm GO, we further evaluated the disinfection efficacy of this combination (Fig. 3). Treatment with 1% AA and 8 ppm GO yielded *S.* Typhimurium, *E. coli* O157:H7, and *L. monocytogenes* counts of 6.18, 6.11, and 6.29 log CFU/g, respectively, which were significantly lower than those in the tap water and control groups. However, on day 7, no significant differences were observed, suggesting that 1% AA + 8 ppm GO was not suitable for controlling foodborne pathogens on fresh-cut lettuce. Additionally, 1% LA, 2% LA, 100 ppm SH, 200 ppm SH, and 8 ppm GO reduced the *S.* Typhimurium counts by 0.78, 0.95, 0.44, 0.74, and 0.59 log CFU/g, respectively (Fig. 2A), whereas 1% LA + GO, 2% LA + GO, 100 ppm SH + GO, and 200 ppm SH + GO reduced these counts to 1.14, 1.28, 0.75, and 0.87 log CFU/g, respectively (Fig. 3A), indicating that synergistic effects did not occur.

Many studies have shown that hurdle technology cannot provide synergistic disinfection effects, but it can enable additional microbial reduction when compared with single disinfection methods [27], [28], [29], [30]. For *E. coli* O157:H7 and *L. monocytogenes*, the disinfection effects of the four combinations were similar to those observed for *S.* Typhimurium (Fig. 3B and C). Treatment with 2% LA + GO yielded the lowest microbial counts for *E. coli* O157:H7 and *L. monocytogenes* (5.41 and 5.71 log CFU/g, respectively); these values were significantly lower than those for SH + GO. The antibacterial activities of organic acids are traditionally attributed to cellular anion accumulation, which is associated with the dissociation constant (p*K*a). Compared with dissociated anions, undissociated acidic molecules have stronger lipophilicity, allowing them to penetrate the microbial cell membrane more easily. After penetration, the higher intracellular pH in the environment promotes dissociation of acid molecules, and the dissociated anions accumulate in the cell and exert toxic effects on DNA, RNA, and ATP synthesis [23], [31]. The antibacterial mechanism of ozone and SH destroys the cell membrane of the target microbe [7], [32], [33]. Thus, the combination of two types of sanitizers with different antibacterial mechanisms can explain the higher disinfection efficacy of 2% LA + GO when compared with that of SH + GO (i.e., oxidizing sanitizer + oxidizing sanitizer).

During storage, the counts of *E. coli* O157:H7, *L. monocytogenes*, and *S.* Typhimurium in the control and tap water groups were not significantly increased, consistent with a previous study [34]. From day 3 to day 7, the counts of *S.* Typhimurium and *E. coli* O157:H7 in the 1% AA + GO group were significantly increased, indicating that this combination could stimulate the growth of *S.* Typhimurium and *E. coli* O157:H7. Similarly, another study found that *L. monocytogenes* was stimulated to grow on lettuce after washing with 0.5% propionic acid (PA), whereas 1% PA significantly reduced the counts of this bacterium. The authors suggested that this result may be explained by the observation that, compared to the native microflora, *L. monocytogenes* is more resistant to 0.5% PA and more competitive, whereas 1% PA can create an acidic environment that exceeds the upper limit of resistance of the bacterium [47]. The disinfection efficacy of organic acids is associated with the p*K*a value; AA has a p*K*a value of 4.75, which is similar to that of PA (4.87). With the increase in storage days, the counts of *E. coli* O157:H7, *L. monocytogenes*, and *S.* Typhimurium in the LA + GO group showed a decreasing trend, and the combination of 2% LA + GO led to the lowest counts on days 3 and 7; these values were significantly lower than those for SH + GO. Similarly, researchers in a previous study [35] found that the counts of *L. monocytogenes* on broccoli showed a decreasing trend during storage after washing with organic acid + oxidizing sanitizer (i.e., LA + SH). In summary, the combination of 2% LA + GO appeared to be the best choice for controlling foodborne pathogens on fresh-cut lettuce.

**Fig. 4:**
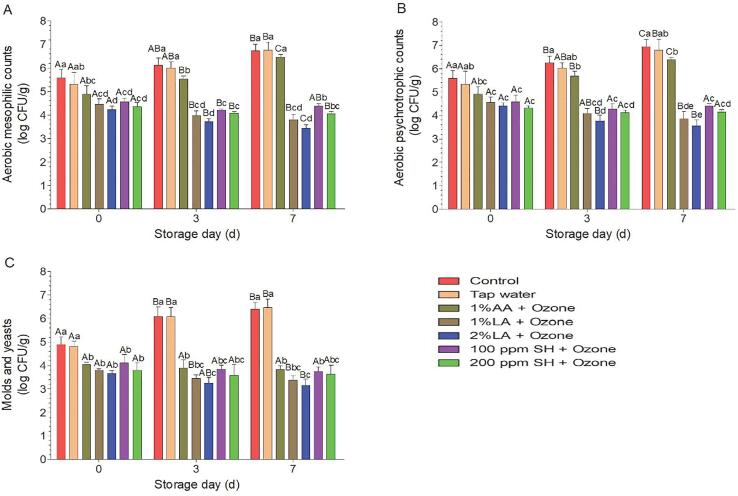
Disinfection effects against aerobic mesophilic bacteria (A), aerobic psychrotrophic bacteria (B), and molds and yeasts (C) during storage. Within the same day, mean values with different lowercase letters are significantly different from each other (P < 0.05); within the same treatment, mean values with different capital letters are significantly different from each other (P < 0.05). AA, acetic acid; LA, lactic acid; SH, sodium hypochlorite. The concentration of ozone was 8 ppm.

For naturally present microbes, among the five treatment combinations, the 2% LA + GO group had the lowest counts (4.23, 4.41, and 3.66 for AMC, APC, and M&Y, respectively) on day 0 (Fig. 4A–C). According to a previous review, the disinfection efficacy against naturally present microbes does not exceed 3 log [17], which is mainly because of embedding of microbial cells into inaccessible parts of irregular produce surfaces [18]. During storage, the AMC, APC, and M&Y counts in the control group showed an increasing trend, consistent with previous studies [10], [20], [34]. For the 1% AA + GO group, the AMC and APC showed an increasing trend, and on day 7, the AMC and APC were nonsignificant in the control group, indicating that 1% AA + GO could stimulate the growth of AMC and APC. From day 3 to day 7, 2% LA + GO treatment significantly reduced the AMC and APC levels compared with those in the SH + GO treatment group. At the end of storage (day 7), the AMC, APC, and M&Y counts in the LA + GO group were significantly lower than those on day 0, whereas those in the SH + GO group were not significantly different from those on day 0. These results indicated that 2% LA + GO was the best choice for controlling microbes natively present in fresh-cut lettuce.

### 3.3 Effects of different combinations on the quality properties of fresh-cut lettuce

#### 3.3.1 Color

**Fig. 5:**
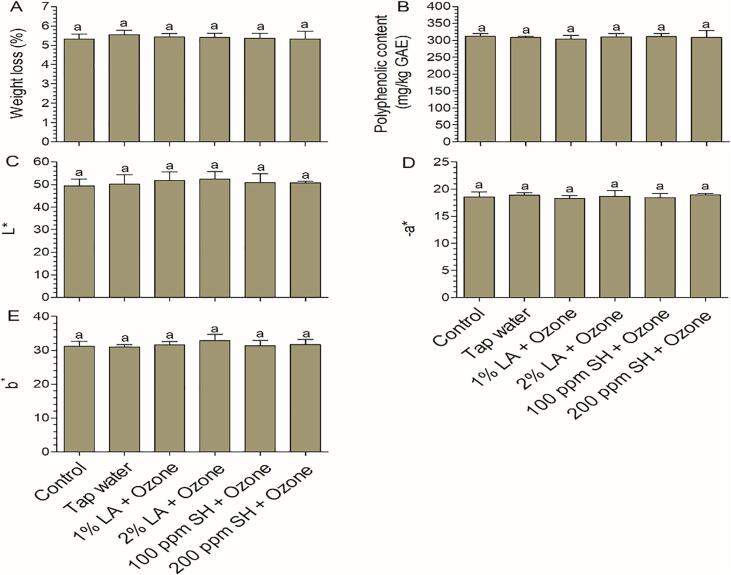
Quality properties at 7 days for fresh-cut lettuce after disinfection with several combinations of treatments. Weight loss (A), polyphenolic content (B), L* (C), a* (D), and b* (E) are shown. Bars show means ± standard deviations, and different letters above the columns indicate significant differences (P < 0.05). AA, acetic acid; LA, lactic acid; SH, sodium hypochlorite. The concentration of ozone was 8 ppm.

Purchase decisions for minimally processed leafy greens are strongly influenced by their color, which directly affects consumer visual perception [34]; thus, it is important to combine evaluations of instrument color with sensory quality. For instrument color, L*, a*, and b* were evaluated, where negative to positive values represent dark to light, green to red, and blue to yellow, respectively. Most types of sanitizers are known to cause damage to vegetable leaves; however, the extent of damage determines its final quality. Because browning spots were observed in samples treated with 1% AA + 8 ppm GO, the data (quality and sensory analysis) were not evaluated for this group. For the other four groups, the value of b* was not significantly different from that of the control group (Fig. 5E). L* and a*, which were used to evaluate whether green leafy vegetables were discolored after washing with sanitizers, of the treated samples were not significantly different from those of the control group (Fig. 5C and D).

Previous studies have shown that GO can cause color deterioration in lettuce leaves. For example, in one study [36], increasing the GO concentration from 2.5 to 4 ppm and processing for 15 min caused the leaves to lose their green color and induced a translucent appearance. In analyses of other types of vegetables, the L* values of carrot slices were found to increase after GO treatment because of the enzymatic reaction causing the formation of lignin [37]. A similar phenomenon was also observed when using aqueous ozone; the visual quality of fresh-cut lettuce deteriorated as the concentration increased from 3 to 10 ppm [38]. Hydrogen peroxide, another type of oxidizing sanitizer, can cause the browning of fresh-cut lettuce, as measured using a* values [39]; this phenomenon was observed when using a high concentration (5%), but not observed using a low concentration (1%) [40]. Oxidizing sanitizers cause tissue deterioration mainly due to their high oxidant power, which results in destruction of leaf tissue and promotion of the enzymatic activity of phenylalanine ammonia lyase [41].

Compared with oxidizing sanitizers, organic acids typically have fewer negative effects on color quality. Bermúdez-Aguirre and Barbosa-Cánovas [36] found that the L* and white indices increased after exposure to 15 ppm GO, whereas 0.5–1.5% citric acid did not interfere with the visual quality of fresh-cut lettuce. Poimenidou et al.[42] reported that the b* values of lettuce after storage for 6 days were 37.1 and 22.5, following washing with 300 ppm FC and 2% LA, respectively. In this study, when the two oxidizing sanitizers were combined (SH + GO), the color quality was not negatively affected. We expect that this result could be explained by the use of appropriate concentrations and treatment times. According to previous studies, when organic acids are combined with oxidizing sanitizers, the color quality of fresh-cut lettuce is not affected, as exemplified by the combination of organic acid with hydrogen peroxide and aqueous ozone [10], [21], [43]. Similarly, in this study, we found that the color properties were not negatively affected when LA was combined with GO.

#### 3.3.2 Polyphenolic content, individual phenolic content, and weight loss

Polyphenolics are important secondary metabolites of plants and function as key nutrients to prevent oxidative damage in the human cells. After 7 days of storage, the polyphenolic content was 312.34 mg/kg GAE in the control group (Fig. 5B), consistent with previous reports [10], [22]. Nonsignificant differences were observed between the combination groups and the control group, indicating that the proposed combinations did not negatively affect the polyphenolic content. A comprehensive study showed that UV-C and GO treatment did not decrease the polyphenolic content; however, the contents of some individual phenolics, such as procyanidins, flavonols, ellagic acid, and pcoumaroyl glucose, were slightly reduced [44]. For some crops with a high content of polyphenolics, such as papaya [45], [46], *Ganoderma lucidum* [47], guava, honey pineapple, and banana [48], GO treatment can increase the polyphenolic content further.

**Fig. 6:**
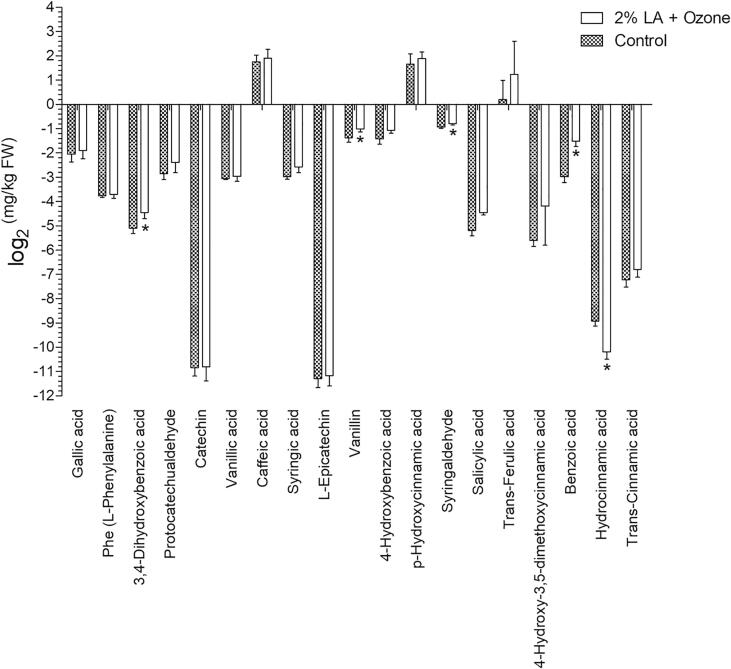
Phenolic acid profile of fresh-cut lettuce at day 7. Bars show mean ± standard deviation values, and the asterisk symbols above the columns indicate significant differences (P < 0.05). LA, lactic acid; FW, fresh weight. The concentration of ozone was 8 ppm.

To perform an in-depth analysis of the phenolic metabolism after treatment, the changes in individual phenolic contents in the control and 2% LA + 8 ppm ozone (because this group showed the highest microbial reduction during storage) groups were compared. As phenolic acids are the major phenolic compounds in lettuce [49], [50], [51], targeted metabolomics, using 19 individual phenolic acids, was employed. The linear regression and quantitation report for each mass peak are shown in Table S1 and S2, respectively. The results indicated that the major individual phenolic compounds were caffeic acid, p-hydroxycinnamic acid, and *trans*-ferulic acid (Fig. 6), which is consistent with previous studies [49], [52]. These phenolic compounds were not significantly changed after treatment with 2% LA + 8 ppm ozone. However, the levels of 3,4-dihydroxybenzoic acid (protocatechuic acid), vanillin, syringaldehyde, benzoic acid, and hydrocinnamic acid (phenylpropanoic acid) were significantly altered after treatment. Among them, 3,4-dihydroxybenzoic acid and vanillin are responsible for biosynthesis of phenylpropanoids, which is responsible for lettuce browning [51]. Similarly, phenylpropanoic acid is responsible for phenylalanine metabolism. Surface damage, as a consequence of disinfection process, is an unavoidable phenomenon. When the extent of damage is large, browning occurs because phenolic substances react with oxygen under the action of polyphenol oxidase. Therefore, the extent surface damage determines the degree of browning. L* and a* can reflect the degree of browning. At the end of storage, L* and a* of the treatment group were similar to those of the control group (Fig. 5C and E), indicating that the lettuce surface was slightly damaged and does not cause significant color changes.

During storage, the weight loss of fresh produce was mainly due to nutrient loss and water segregation [53]. Transformation from phenolics to quinones (key markers of browning) and segregation of water are accelerated as the extent of sanitizer-induced damage increases. In this study, weight loss in the control group on day 7 was 5.33% (Fig. 5A), and similar results were obtained for the other combination groups. These findings suggested that there was only minor damage caused by the different combinations, leading to self-repair of the leaf tissue in subsequent storage, without causing phenolic loss and weight loss [10], [54].

#### 3.3.3 Sensory quality

**Fig. 7:**
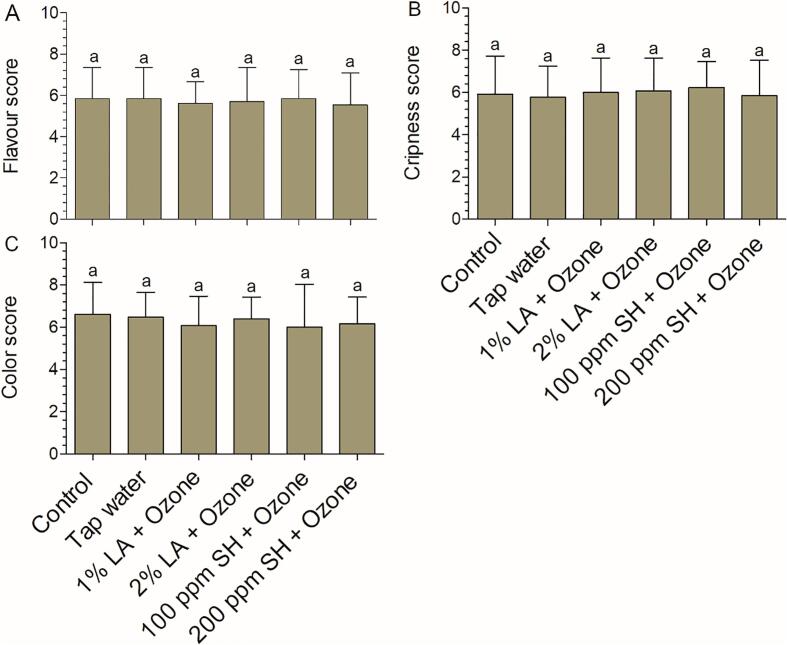
Sensory quality of fresh-cut lettuce at 7 days after disinfection with several combinations. Flavor (A), crispness (B), and color (C) scores are shown. Bars show mean ± standard deviation values, and different letters above the columns indicate significant differences (P < 0.05). AA, acetic acid; LA, lactic acid; SH, sodium hypochlorite. The concentration of ozone was 8 ppm.

Sensory color, flavor, and crispness are crucial factors affecting consumer acceptance [20], [25], [44]. The sensory evaluation results are shown in Fig. 7. At the end of storage (7 days), the sensory color score in the combination groups was not significantly different from that in the control group (Fig. 7C), consistent with the results of instrument color analysis (Fig. 5C–E). Previous studies have shown that the crispness of fresh-cut lettuce is not affected by combination of an oxidizing sanitizer with organic acids (e.g., hydrogen peroxide + citric acid and LA + aqueous ozone) or an oxidizing sanitizer with another oxidizing sanitizer (e.g., hydrogen peroxide + electrolyzed water and chlorine + aqueous ozone) [10], [21]. Similarly, in our study, crispness was not affected by the four combinations (Fig. 7B). Lettuce is generally eaten raw, and in this study, our analysis indicated that the four combinations did not negatively affect the sensory flavor (Fig. 7A).

## 4 Conclusion

In this study, we proposed a non-immersion disinfection method for lowering sanitizer dosage and the risk of cross-contamination. Aerosolized sanitizer and GO were combined to disinfect fresh-cut lettuce. Our results showed that 2% AA and 1% AA + 8 ppm GO could cause browning of lettuce leaves, and 1% AA and 4 ppm GO did not significantly reduce *S.* Typhimurium levels. Additionally, 1% LA + 8 ppm GO, 2% LA + 8 ppm GO, 100 ppm FC + 8 ppm GO, and 200 ppm FC + 8 ppm GO did not negatively affect the quality and sensory properties of fresh-cut lettuce. Overall, LA + GO and SH + GO could significantly disinfect foodborne pathogens (i.e., *E. coli* O157:H7, *L. monocytogenes*, and *S.* Typhimurium) and naturally present microbes (i.e., AMC, APC, and M&Y), with 2% LA + 8 ppm GO resulting in the lowest counts. This additional microbial reduction caused by LA + GO may be due to the different antibacterial mechanisms of action of LA and GO (intracellular and extracellular effects, respectively). After LA penetrates the cell membrane, the higher intracellular pH environment promotes its dissociation, which leads to inhibition of DNA, RNA, and protein synthesis; additionally, ozone oxidizes the outer cell membrane. Similarly, SH destroys the cell membrane; thus, the additional microbial reduction caused by SH + ozone may be attributed to the accelerated destruction of cell membrane. In future studies, omics technology should be applied to analyze the changes in bacterial biological processes. Furthermore, scanning electron microscopy, propidium iodide staining, K^+^ leakage analysis, and protein leakage analysis should be carried out to reveal the mechanism of action of the additional microbial reduction caused by LA + GO and SH + GO. Finally, our study showed that 1% AA + 8 ppm GO stimulated increases in AMC, APC, *S.* Typhimurium, and *E. coli* O157:H7. These findings provided important insights into the use of organic acids combined with GO for application in disinfecting fresh produce.

## CRediT authorship contribution statement

**Jiayi Wang:** Conceptualization, Supervision, Funding acquisition, Writing - original draft, Writing - review & editing. **Yangyang Zhang:** Investigation, Methodology, Writing - original draft. **Yougui Yu:** Writing - review & editing. **Zhaoxia Wu:** Data curation. **Hongbin Wang:** Formal analysis.

## Declaration of Competing Interest

The authors declare that they have no known competing financial interests or personal relationships that could have appeared to influence the work reported in this paper.
